# Efficacy of palifermin in the treatment of oral mucositis in non-hematopoietic stem cell transplant pediatric patients: experience of a single tertiary hospital

**DOI:** 10.1186/s43046-024-00247-x

**Published:** 2024-12-02

**Authors:** Hasna Hamzi, Amal Binhassan, Amal Najmeldin, Ebtsam Alhariri, Bodour Elhussein, Nour Althibani, Ahmad Alenazi, Shahad Alsharif, Mohammad Alshahrani, Omar Alsharif, Nawaf Alkhayat, Yasser Elborai

**Affiliations:** 1 Department of Pediatric Hematology/Oncology, Prince Sultan Military Medical City, Riyadh, Saudi Arabia; 2 Department of Pediatric, Alyamamah Hospital, Riyadh, Saudi Arabia; 3 Department of Pediatric Oncology, National Cancer Institute, Cairo University, Cairo, Egypt

**Keywords:** Palifermin, Oral mucositis, Pediatric malignancy, Hematopoietic stem cell transplant

## Abstract

**Background:**

Severe oral mucositis (OM) is one of the adverse events post-chemotherapy, radiation, and stem cell transplantation with major clinical and economic impact. The management of severe OM remains challenging. This study aimed to look for the benefit and clinical impact of palifermin for mucositis among the non-transplanted pediatric cancer population.

**Methods:**

This is a descriptive retrospective study extended from 2016 to 2020 at Prince Sultan Military Medical City (PSMMC), in Saudi Arabia. During this period all pediatric patients (< 14 years of age) on chemotherapy and complicated by severe OM that required palifermin (35 courses), as off-labeled medication, were analyzed looking for the clinical demographics, primary diagnosis, chemotherapy agents used, effectiveness, and tolerability of palifermin.

**Results:**

A total of 29 patients with severe OM received 35 palifermin courses. All of them received 60 mcg/kg/day IV for 3 consecutive days. 20% of them have acute lymphoblastic leukemia (ALL). We noticed that 60% of severe OM required palifermin post anthracycline while high-dose methotrexate aggravates the risk in 40% of them. Only 25.7% of the patients required TPN for a median duration of 5 days and 54.3% of them received opioids for a median duration of 4 days. Twenty patients (57.1%) had used antibiotics, 4 patients were on antifungal medication and 1 patient was on anti-viral medication concomitant with antibiotics.

**Conclusion:**

Palifermin is safe and well tolerated and shows some effect in non-hematopoietic stem cell transplant pediatric patients with severe oral mucositis post-intensified chemotherapy or radiation therapy.

## Background

Oral mucositis (OM) is one of the most common and serious side effects of chemotherapy, radiation, and stem cell transplantation; this side effect makes it challenging to manage patients with hematological and non-hematological malignancies [1]. Furthermore, OM has a significant effect on patients’ health and quality of life [2] as well as a major economic impact on health institutes [3].

Chemotherapy that interferes with DNA synthesis, repair, and cellular replication (S-phase-specific agents such as methotrexate, cytarabine, and fluorouracil) seems to be more stomatotoxic in nature [1]. It is more pronounced with boluses and continuous infusions than with a small, frequent dose of the same medication [4].

Severe OM has important clinical implications, as it may be a risk factor for chemotherapy delay, dose reduction, or frequent interruption of chemotherapy cycles, which may lead to compromised management and increased risk of treatment failure or relapse in pediatric patients with malignancies [2].

Palifermin is a recombinant keratinocyte growth factor (KGF) that was initially established as a human treatment for patients undergoing head and neck chemoradiation in phases I and II, and it is now widely used [5]. It works by activating the NF-E2 p45-related factor 2 (NRF2) pathway, which prevents DNA strand cleavage and stimulates antiapoptotic factors such as Bcl-2 and p53, which play important roles in epithelial regeneration [1, 6].

Here, at Prince Sultan Military Medical City (PSMMC) in the Pediatric Oncology Department, palifermin has been used since 2016 for non-hematopoietic stem cell transplantation in both hematological and non-hematological malignancy pediatric patients. Given that palifermin studies in the pediatric population, especially non-hematopoietic stem cell transplantation groups, are limited, we conducted this retrospective study to explore the effects of the use of palifermin on the severity of OM, duration, and supportive care in pediatric patients who suffered from severe OM after chemotherapy between 2016 and 2020.

## Methods

### Study population

This descriptive retrospective study was extended from 2016 to 2020 at the PSMMC. During this period, all pediatric patients on chemotherapy complicated by severe OM that required palifermin (60 mcg/kg/day for 3 consecutive days) were identified, and their clinical courses were reviewed. Mucositis definition and grading were performed based on common terminology criteria for adverse events (CTCAE version 5). Parents’ acceptance and signatures were collected before drug administration as per hospital policy in form B, as the medication was prescribed as off-label. Institutional review board (IRB) approval was obtained, and the data were reviewed in a password-protected electronic database in addition to paper files.

### Statistical analysis

Patient characteristics and prescribing patterns of palifermin are reported via descriptive statistics. All analyses were performed via the Statistical Package for Social Sciences (SPSS) version 23.0 (SPSS Inc., IBM, Armonk, NY, USA). The results are expressed as numbers and percentages for categorical variables and as the means, medians, and standard deviations for continuous variables. Comparisons between two continuous means were performed via independent *t* tests and analysis of variance (ANOVA) for three or more means. Chi-square tests were used to compare the two groups. A *p* value of < 0.05 was considered to indicate statistical significance.

## Results

### Patient characteristics

In this retrospective study, we evaluated 29 patients; specifically, 23 patients received only one course, and 6 patients received 2 courses, for a total of 35 palifermin courses. Only 2 patients out of 35 were less than 2 years old, whereas 16 and 17 patients were 3–10 years old and older than 10 years old, respectively. The male:female ratio was 2:1. A total of 28/35 patients (80%) had grade 3 toxicity, whereas 7/35 patients (20%) had grade 4 toxicity (Table 1).

**Table 1:** Characteristics of patients who received palifermin

	*N* = 35	Percentage %
Age:		
< 2 years old	2	5.7
3–10 years old	16	45.7
> 10 years old	17	48.6
Gender:		
Male	23	65.7
Female	12	34.3
Grade of mucositis:		
3	28	80.0
4	7	20.0
Primary diagnosis:		
ALL	7	20.0
Relapsed ALL	6	17.1
Non-Hodgkin lymphoma	6	17.1
AML	4	11.4
Hodgkin lymphoma	2	5.7
Neuroblastoma	3	8.6
Osteosarcoma	3	8.6
Rhabdomyosarcoma	3	8.6
Ewing’s sarcoma	1	2.9
Chemotherapy agents/radiation:		
High-dose methotrexate	14	40.0
High-dose cytarabine	1	2.9
Anthracycline	21	60.0
Cyclophosphamide or ifosfamide	17	48.6
Methotrexate	3	8.6
Etoposide	10	28.6
Bleomycin	2	5.7
High-dose methotrexate and cyclophosphamide/ifosfamide	8	22.9
High-dose methotrexate and anthracycline	8	22.9
Cytarabine and anthracycline	4	11.4
Anthracycline and (cyclophosphamide or ifosfamide)	13	37.1
Anthracycline and alkylating agents and high-dose methotrexate	7	20.0
Radiation therapy	2	5.7

### Primary disease/chemotherapy

All leukemia/lymphoma patients were in complete remission during OM episodes (25/35). The solid tumor patients who experienced 10/35 episodes of mucositis followed the treatment protocols smoothly in different phases (Table 1). All the episodes of mucositis occurred during the neutropenic phase after chemotherapy and radiotherapy. The most important chemotherapies that cause mucositis are anthracycline, cyclophosphamide/ifosfamide, and high-dose methotrexate. Many other chemotherapy agents can cause mucositis at a relatively low percentage; however, patients may receive more than one agent per cycle, so we cannot precisely identify which agent is the causal agent. Only 2 patients suffered from mucositis after radiation (Table 1).

### Efficacy and tolerability

All the patients received 60 mcg/kg/day IV palifermin for the management of Grade III and IV mucositis for 3 consecutive days. It was tolerable, and no adverse events were documented. The median duration of complete resolution of mucositis was 5 days (range 2–10 days). Nineteen out of 35 patients (54.3%) required opioids for a median duration of 4 days (range 2–7 days). Nine out of 35 patients (25.7%) required total parental nutrition (TPN) for a median duration of 5 days (range 4–10 days). Twenty out of 35 patients (57.1%) received antibiotics for a median duration of 7 days (range 5–14 days); four of them received antifungal treatment concomitant with antibiotics; one patient received antiviral treatment concomitant with antibiotics; and one patient had a positive culture and was thus admitted to the intensive care unit (ICU) (Table 2).

**Table 2:** Supportive care post-palifermin

	*N* = 35	Percentage %	Median (range) days
Opioid			
Yes	19	54.3	4 (2–7) days
No	16	45.7	
TPN			
Yes	9	25.7	5 (4–10) days
No	26	74.3	
Antibiotic			
Yes	20	57.1	7 (5–14) days
No	15	42.9	

## Discussion

OM induced by chemotherapy, radiation, or stem cell transplantation may interfere with the patient’s treatment plan, which may affect the outcome [3]. The use of palifermin can mitigate, to a certain extent, the outcome in pediatric cancer patients complicated by severe mucositis after intensive chemotherapy/radiotherapy in terms of duration, severity, and clinical sequelae [7].

In our study, 40% of our patients with severe mucositis received high-dose methotrexate, 60% received anthracycline, 48.6% received alkylating agents (cyclophosphamide or ifosfamide), and two patients (5.7%) developed mucositis after radiotherapy. Al-Ansari et al. reported that a large proportion of patients who develop OM receive high-dose chemotherapy-based conditions prior to stem cell transplantation (approximately 75–80%) [8].

In our study, 25/35 patients (71.4%) developed OM after chemotherapy for leukemia/lymphoma, whereas 10 patients (28.6%) developed OM after chemotherapy/radiotherapy for solid tumors. OM was grade 3 and grade 4 in 80% and 20% of patients, respectively. Vera-Llonch et al. reported that 51% of patients (303/599) developed oral and/or gastrointestinal mucositis after receiving chemotherapy for lymphoma or solid tumors. Among 1236 cycles of chemotherapy, 22% of patients suffered from OM, 7% from GI mucositis, and 8% from both oral and GI mucositis [9].

Many strategies are used to minimize the severity of OM and its sequelae [10, 11]. Maintaining good oral hygiene is the most important step in management [12]. In addition, many studies have shown the benefits of using GCSF in solid tumors, as it helps reduce the duration of neutropenia and, in turn, reduces mucositis severity and duration [6]. All our patients with solid tumors started GCSF 24 h after chemotherapy according to the protocol.

Starting IV fluids and TPN, as soon as symptoms of OM arise, is essential to maintain optimum nutritional health while keeping the patient sufficiently pain-free by administering opioids [13, 14]. Occasionally, antibiotics, antifungals, and antivirals are needed [15]. Although care for OM is supportive, its management is still challenging due to its complexity [16].

In our study, more than half of our patients (19/35 [54.3%]) used opioids for a median duration of 4 days (range 2–7 days). However, TPN was utilized in only 9/35 patients (25.7%), with a median duration of 5 days (range 4–10 days). The durations of opioid use and TPN in our patients were comparable to those reported by Spielberger et al., who reported a shorter opioid duration (7 days) and TPN administration rate of 31% in the palifermin group, whereas a longer opioid duration (11 days) and TPN administration rate of 55% were reported in the placebo group [17].

In 2016, a randomized controlled study by Alessandra Lucchese et al. showed promising results in reducing the opioid dose, the duration of TPN administration and, ultimately, the hospitalization duration when palifermin was used as prophylaxis during hematopoietic stem cell transplant (HSCT) therapy for pediatric patients with acute lymphoblastic leukemia to reduce OM [18].

Another study performed by Dazhi et al. in 2017 reported that Alessandra Lucchese et al. used palifermin as secondary prophylaxis. This retrospective study included 18 pediatric patients with a history of chemotherapy-induced mucositis (non-HSCT) who received palifermin as secondary prophylaxis, which yielded promising results. Specifically, 72% of the patients in the palifermin group did not develop mucositis, the opioid requirement was decreased in 68%, and the duration of hospitalization was reduced in 11 out of 18 patients. Only 10 patients were started on antibiotics because all blood cultures were negative after palifermin [7].

Severe mucositis is related to acute inflammation of the mucous membrane after chemotherapy or radiation, which leads to disruption of the integrity of the normal mucosa and increases the risk of infection, resulting in systemic sepsis, mainly during immunosuppression [19]. Our study revealed that only 20 out of 35 patients required systemic antibiotics, despite the absence of positive cultures or ICU admissions, except in 1 patient.

Czyzewski et al. reported a lower incidence of fever and sepsis in post-HSCT patients who received palifermin [20]. In addition, Spielberger et al. reported lower febrile neutropenia (75% vs. 92%) and lower sepsis (15% vs. 25%) rates among palifermin recipients compared with placebo patients [17].

Although there are few studies on the use of palifermin in pediatric patients, they have proven its safety, with no significant complications [5, 21]. Our data indicate that palifermin administration was tolerable without any documented adverse effects among our patients during treatment.

## Conclusion

This study demonstrated that palifermin utilization is safe and well tolerated and has some effects on non-hematopoietic stem cell transplant pediatric patients with severe OM after intensified chemotherapy or radiation exposure, which could affect patient quality of life and health economics. Our study has several limitations, including its small sample size, single-center nature, and lack of randomization and control groups. Prospective studies in the future are needed to assess the role of palifermin initiation in patients with severe mucositis after chemotherapy, identify appropriate parameters for indications based on clinical criteria and chemotherapy type and dose, and consider it as prophylaxis in certain cases in the future.

## Data Availability

The data that support the findings of this study are available from a password-protected database in Prince Sultan Military Medical City, Riyadh, Saudi Arabia. However, restrictions apply to the availability of these data, which were used under license for the current study, and so are not publicly available. Data are however available from the authors upon reasonable request and with permission of Prince Sultan Military Medical City.

## Abbreviations

OM: Oral mucositis
KGF: Keratinocyte growth factor
NRF2: NF-related factor 2
PSMMC: Prince Sultan Military Medical City
ICU: Intensive care unit
CTCAE: Common terminology criteria for adverse events
IRB: Institute review board
SPSS: Statistical Package for Social Sciences
TPN: Total parental nutrition
GCSF: Granulocyte colony-stimulating factor
HSCT: Hematopoietic stem cell transplant
